# Acute Right Ventricular Failure in a Patient with Hepatic Cirrhosis

**DOI:** 10.1155/2012/127583

**Published:** 2012-12-18

**Authors:** Jose Soto Soto, Xochiquetzal Geiger, Margaret M. Johnson

**Affiliations:** ^1^Division of Pulmonary Medicine, Mayo Clinic Florida, Jacksonville, FL 32224, USA; ^2^Department of Laboratory Medicine and Pathology, Mayo Clinic Florida, Jacksonville, FL 32224, USA

## Abstract

Pulmonary embolic disease is most commonly a manifestation of venous thromboembolism (VTE). However, fat, tumor, and air may all embolize to the pulmonary vasculature and lymphatics resulting in various clinical manifestations. Tumor emboli to small pulmonary vessels and lymphatics can lead to hypoxemic respiratory failure and shock. We present a 62-year-old male with history of mild COPD and end-stage liver disease secondary to hepatitis C admitted due to progressive shortness of breath and hypoxemia who developed shock and right ventricular failure. After a negative evaluation for venous thromboembolic disease, he had progressive respiratory and hemodynamic deterioration despite mechanical ventilation, renal replacement therapy, and vasopressive/inotropic support. Postmortem examination revealed diffuse micronodular moderately differentiated hepatocellular carcinoma (HCC) without a discrete mass, as well as numerous HCC tumor emboli to the lung and focally to the heart, consistent with disseminated hepatocellular tumor microembolism.

## 1. Introduction

Disseminated pulmonary tumor microembolism is an uncommon manifestation of metastatic cancer. It is due to obstruction of the pulmonary vasculature by tumor microemboli with resultant pulmonary hypertension, right ventricular failure, and cor pulmonale. This diagnosis must be considered in a patient with risk factors for malignancy in the setting of acute hypoxemic respiratory failure and rapidly worsening right ventricular systolic function without evidence of venous thromboembolism. 

## 2. Case Report

**Figure 1:**
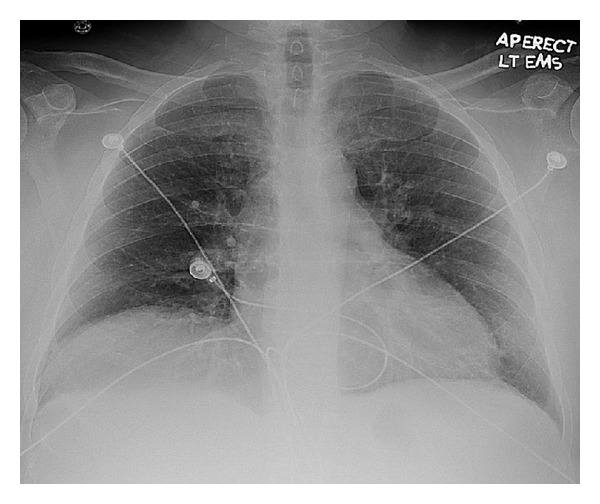
Increased interstitial markings bilaterally and right hemidiaphragm elevation.

**Figure 2:**
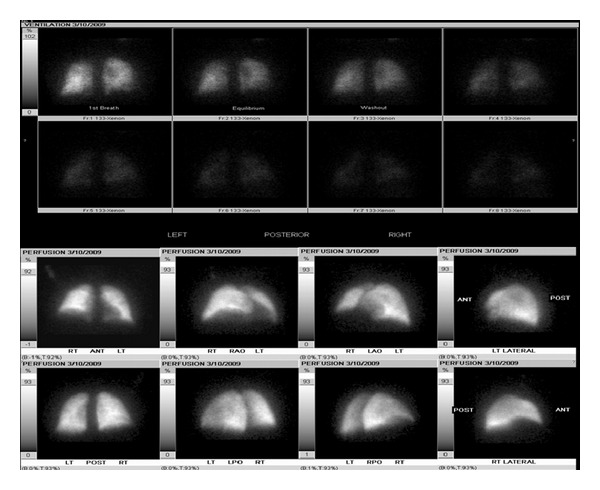
Low probability for venous thromboembolism.

**Figure 3:**
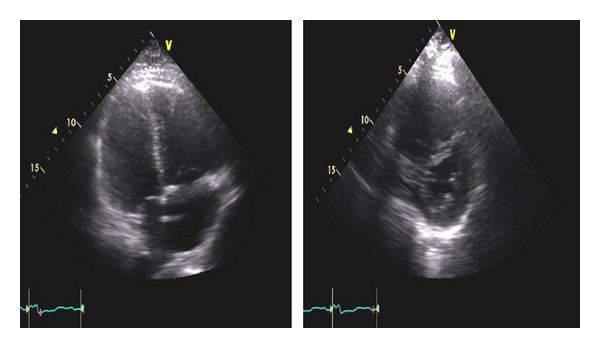
Systolic apical 4-chamber view with normal-sized left ventricle on the left and markedly dilated right ventricle on the right, (b) Parasternal short-axis view of left ventricle shows “D-shaped” left ventricle indicating right ventricular pressure overload and markedly dilated and thin-walled right ventricle on the top.

**Figure 4:**
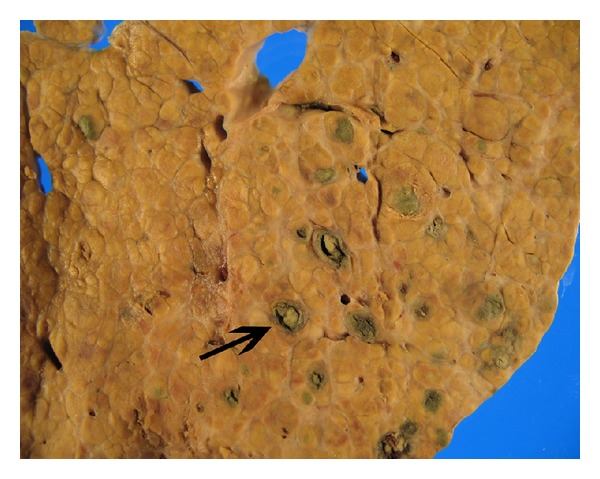
Cirrhotic liver with numerous HCC micronodules (arrow).

**Figure 5:**
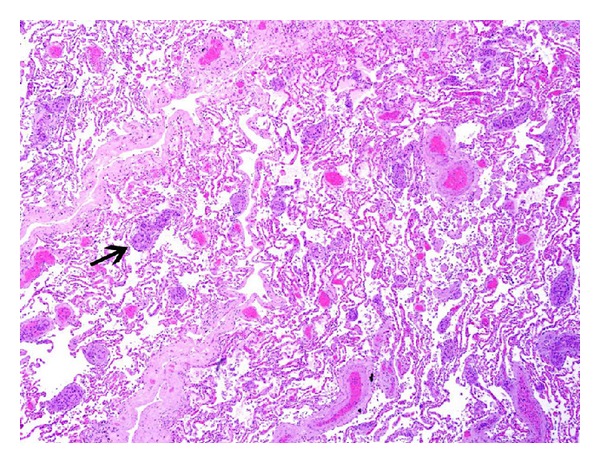
Numerous capillary and lymphatic HCC tumor emboli (arrow).

**Figure 6:**
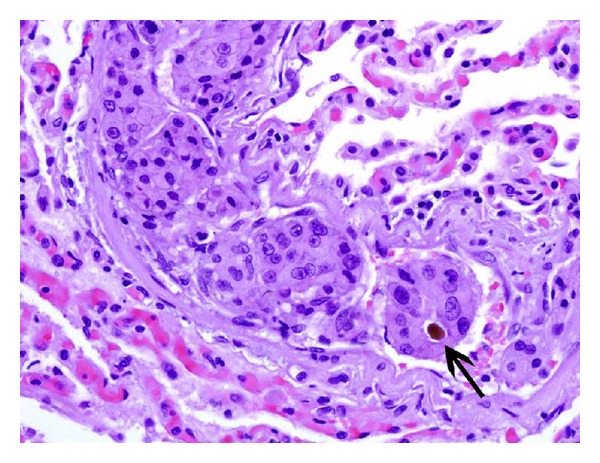
Lung tissue with intravascular tumor. Focal bile consistent with HCC (arrow).

A 62-year-old male with a past history of COPD, hepatitis C infection, end-stage liver disease (MELD score 15), esophageal varices, and diabetes mellitus type II was admitted for progressive shortness of breath of one-week duration. Exam was notable for normothermia, heart rate 95 bpm, respirations 21 bpm, blood pressure 134/78, and oxygen saturation of 85% on RA; cardiopulmonary exam was remarkable for regular rate and rhythm, no murmurs, JVD 3 cm at 30 degree angle, nonpalpable PMI or right ventricular lift; lung exam showed diffuse expiratory wheezing without crackles or rhonchi. The remainder of the examination was unremarkable, including strong peripheral pulses and no edema or cyanosis. Chest X-ray showed no changes of hyperinflation or parenchymal infiltrates, small bilateral pleural effusions, and right hemidiaphragm elevation (Figure 1). Arterial blood gas showed a pH of 7.45, PaCO2 of 35.9 mm Hg, and PaO2 of 59 mm Hg. The respiratory alkalosis and A-a gradient raised concerns for venous thromboembolism (VTE), but lower extremity ultrasound was negative for thrombus. CT angiography was not performed due to renal dysfunction (Cr 4.7 mg/dL). A ventilation/perfusion scan showed low probability for VTE (Figure 2). On the second hospital day, he developed progressive hypotension and syncope in conjunction with worsened hypoxemia. A computed tomography of the head was negative for acute CVA or subdural hematoma. Echocardiography revealed a D-shaped left ventricle with normal systolic function, LVEF 65% (Figure 3). The right ventricle was moderately enlarged with decreased systolic function, but no tricuspid valve dysfunction. Over the subsequent 24 hours, the patient developed severe hypotension unresponsive to fluid resuscitation. Dobutamine therapy was instituted. Repeat echocardiography revealed a small left ventricular cavity (LVEF 50%) with progressive dilation and hypokinesis of the right ventricle and severe tricuspid regurgitation. Supportive measures including mechanical ventilation, vasopressors, and renal replacement therapy were instituted, but refractory shock persisted. The patient developed asystolic arrest. Further resuscitation was withheld in accordance with his family's wishes and the patient expired 72 hrs after admission. Postmortem examination revealed moderately differentiated hepatocellular carcinoma (HCC) with involvement of all liver segments and innumerable tumor micronodules similar in size to the cirrhotic nodules but no dominant HCC lesion. There was invasion of the perihepatic vasculature including the portal vein. There were focal intravascular HCC tumor emboli to the right and left heart ventricles. Although no gross pulmonary emboli were seen, numerous small vessel and lymphatic metastatic HCC tumor microemboli involved all lung lobes (Figures 4, 5, and 6).

## 3. Discussion

Disseminated pulmonary microembolism is due to diffuse metastatic infiltration and obstruction of small vessels and lymphatics by tumor, such as breast, lung, pancreas, prostate, colon, and gastric malignancies [1, 2]. Although it is recognized in <10% of all pulmonary metastases ante-mortem, lymphangitic spread is present in 15% of the autopsies of patients with solid tumors [3]. Adenocarcinomas account for 80% of disseminated pulmonary microemboli and lymphangitic carcinomatosis. As aforementioned, the most common primary tumors are breast, pancreas, prostate, colon, and gastric malignancies [1, 2]; nevertheless, it can also be a consequence of primary pulmonary carcinoma, especially small-cell carcinoma and adenocarcinoma [4]. In most cases of lymphangitic pattern carcinomatosis, pleural involvement is also common. Hypoxia is a very common finding resulting from small vessel tumor emboli and blood vessel compression by distended lymphatics [5]. Acute respiratory failure and/or right-sided heart failure are also common presentations, due to pulmonary hypertension (cor pulmonale) [6]. Occasionally, these may be the presenting features of occult malignancy; thus, this entity must be considered in the differential diagnosis even in the absence of a known primary [2]. Physical examination findings are nonspecific with tachypnea, tachycardia, and diffuse crackles commonly present. Clinical symptoms often precede radiological abnormalities. Radiographic analysis by plain film shows a lower lobe predominant reticulonodular pattern; nevertheless, it can be completely normal [7]. Kerley B lines can be seen without any features of congestive cardiac failure, but all 3 types of Kerley lines can be seen [8]. High-resolution chest CT (HRCT) findings can include circumferentially thickened and irregular or nodular bronchovascular bundles. Transbronchial biopsy has been suggested for diagnosis [9]. Disseminated small vessel tumor microembolism, including patterns of lymphangitic carcinomatosis, carries a very poor prognosis. Reported mean survival is 2 months [10], although 14-month [11] survival has been described. Although commonly used systemic chemotherapy is typically ineffective. The utility of nebulized chemotherapy [12] is being investigated [13].
